# How to treat remnant cholecystitis after subtotal cholecystectomy: two case reports

**DOI:** 10.1186/s40792-021-01183-x

**Published:** 2021-05-03

**Authors:** Taisei Teshima, Hidetoshi Nitta, Chisho Mitsuura, Yuta Shiraishi, Kazuto Harada, Kenji Shimizu, Ryuichi Karashima, Toshiro Masuda, Katsutaka Matsumoto, Tetsuya Okino, Hiroshi Takamori

**Affiliations:** Department of Surgery, Saiseikai Kumamoto Hospital, 5-3-1, Chikami, Minami-ku, Kumamoto, Japan

**Keywords:** Case report, Remnant cholecystitis, Subtotal cholecystectomy, Endoscopic gallbladder drainage

## Abstract

**Background:**

Subtotal cholecystectomy in patients with severe acute cholecystitis is considered a “bailout” option when the safety of the bile duct cannot be guaranteed. However, subtotal cholecystectomy has a long-term risk of remnant cholecystitis. The appropriate management of remnant cholecystitis has not been fully elucidated.

**Case presentation:**

Case 1 was a 66-year-old man who had undergone subtotal cholecystectomy 14 years prior to the development of remnant cholecystitis. We first performed endoscopic gallbladder drainage to minimize inflammation, and then proceeded with elective surgery. We performed a reconstituting procedure for the residual gallbladder due to significant adhesions between the cystic and common bile ducts. Case 2 was a 56-year-old man who had undergone subtotal cholecystectomy for abscess-forming perforated cholecystitis 2 years prior to the development of remnant cholecystitis. He underwent endoscopic drainage followed by complete remnant cholecystectomy 4 months later.

**Conclusion:**

Endoscopic gallbladder drainage is a useful strategy to improve inflammation and reduce the risk of bile duct injury during remnant cholecystectomy.

## Introduction

Laparoscopic cholecystectomy (LC) is the gold standard strategy for acute cholecystitis. The updated Tokyo 2018 Guidelines (TG 18) for the management of acute cholecystitis indicate that acute cholecystitis of Grade III is an indication for LC and should be performed by experienced surgeons at advanced centers [1]. The expanded indication for emergency LC in acute cholecystitis has led, however, to operative challenges, particularly the severe inflammation in the Calot triangle [1]. TG 18 proposed three methods of bailout procedure for operatively difficult cases: subtotal cholecystectomy, conversion to laparotomy, and the fundus first technique. Subtotal cholecystectomy is a particularly useful technique as a bailout surgery to avoid bile duct injury when a critical view of safety cannot be achieved due to severe inflammation at the gallbladder neck [2]. Partial retention of the gallbladder, however, has a long-term risk of recurrence of cholecystolithiasis and remnant cholecystitis [3]. We report two cases of residual cholecystectomy for remnant cholecystitis after subtotal cholecystectomy.

## Patients

### Case report #1

**Fig. 1:**
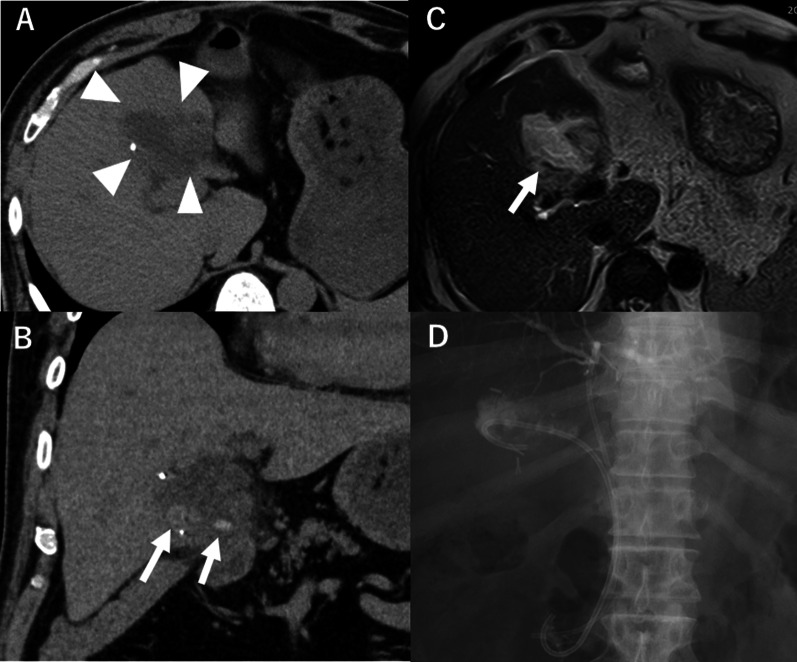
Case 1. **a** Plain abdominal computed tomography. 38 × 36 × 35 mm remnant gallbladder was found (arrowhead), **b** Gallstone was found in the remnant gallbladder (arrow). The fat tissue concentration around the remnant gallbladder was elevated. **c** T2-weighted MR imaging identified the remnant gallbladder with fluid collection around it (arrow). **d** EBS and EGBD tube were placed in the common bile duct and the remnant gallbladder by ERCP, respectively

A 66-year-old male was admitted to the emergency department in our hospital for evaluation of fatigue and shaking chills. He had undergone laparoscopic subtotal cholecystectomy 14 years prior necessitating mesh repair of a ventral hernia one year after. His medical history was notable for type 2 diabetes mellitus and chronic renal failure. Physical examination upon admission revealed mild jaundice and severe epigastric abdominal tenderness. Laboratory testing revealed elevation of inflammatory markers and hepatobiliary enzymes (white blood cell count, 7300 /μL; C-reactive protein, 5.48 mg/dL; aspartate transaminase, 66 IU/L; alanine transaminase, 103 IU/L; alkaline phosphatase, 1456 IU/L; and total bilirubin, 8.8 mg/dL). Abdominal computed tomography (CT) identified a dilated remnant gallbladder (Fig. 1a). A small stone was found in the remnant gallbladder (Fig. 1b). T2-weighted magnetic resonance image (MRI) showed fluid collection around the remnant gallbladder (Fig. 1c). Therefore, we diagnosed this patient with remnant cholecystitis, liver abscess, and Mirizzi syndrome.

Pre-procedure antibiotics (cefoperazone-sulbactam [2 g × 5 days]) were administered, followed by endoscopic biliary stent (EBS) and endoscopic gallbladder drainage (EGBD) tube placement (Fig. 1d). After stent placement and drainage, the patient’s inflammatory markers improved with a white blood cell count of 6100 /μL and C-reactive protein 0.11 mg/dL. The patient returned 5 months later for residual cholecystectomy.

**Fig. 2:**
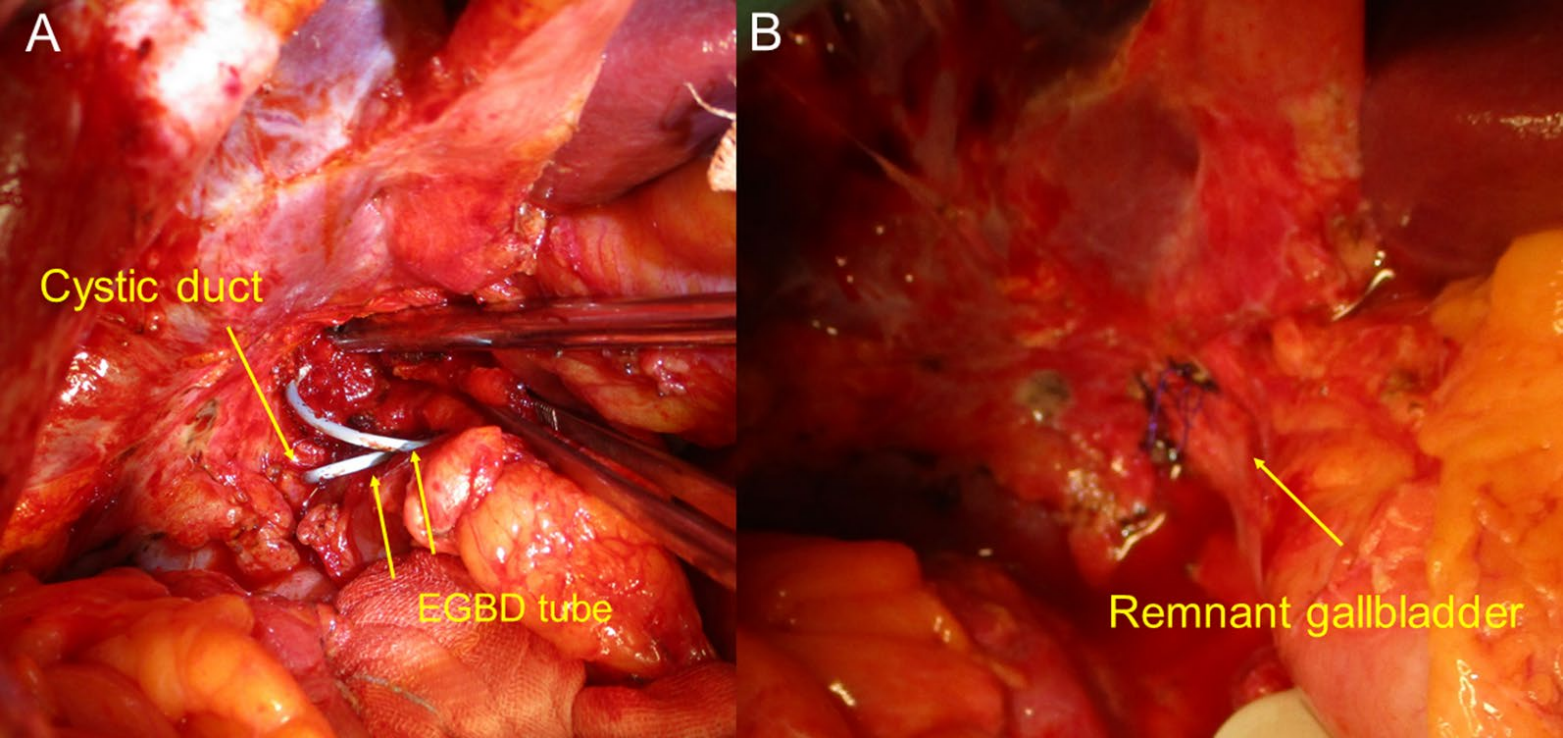
Intraoperative images of case 1. **a** After opening the wall of the remnant gallbladder, the EGBD tube was identified. **b** The remnant gallbladder wall was reconstituted

The laparotomy was carried out via an upper abdominal midline incision cutting the mesh in the abdominal wall. We were able to identify the remnant gallbladder by palpating the EGBD tube, although there were severe adhesions around the liver hilum. The adhesions between the lower part of the remnant gallbladder and the common hepatic duct were difficult to dissect, therefore we performed a subtotal resection of the remnant gallbladder. We opened the remnant gallbladder and removed stones and the EGBD tube (Fig. 2a), then sutured the cystic duct via the fenestrating procedure. We also reconstituted the gallbladder wall after cauterization of the remaining gallbladder mucosa with an electrocautery knife (Fig. 2b). The operative time and blood loss were 231 min and 140 ml, respectively.

The patient did not experience remnant cholecystitis during 9 months of follow-up post-operatively.

### Case report #2

**Fig. 3:**
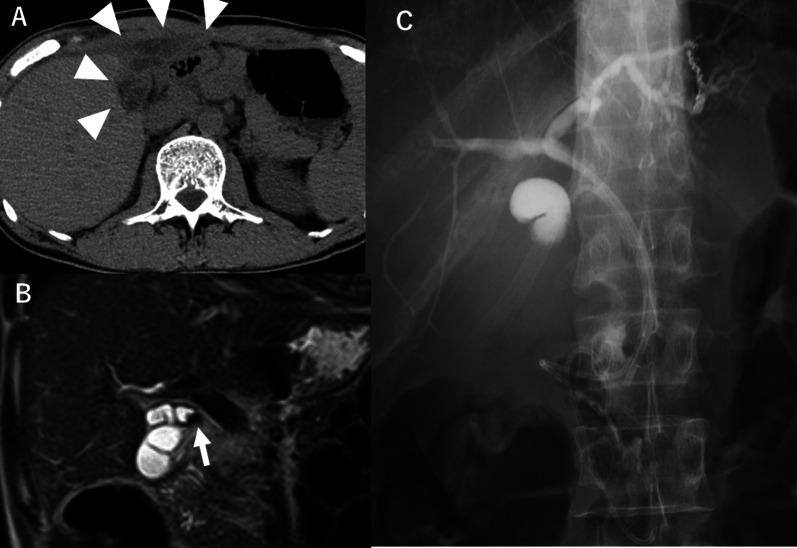
Case 2. **a** Plain abdominal computed tomography showed remnant gallbladder (47 × 29 × 28 mm) with intraabdominal abscess under the abdominal wall (arrow head). **b** A small stone was found in the cystic duct (arrow). **c** The EBS and EGBD tubes were placed in the common bile duct and the remnant gallbladder by ERCP, respectively

A 56-year-old male was admitted to the emergency department of our hospital because of persistent abdominal pain. He had undergone an open subtotal cholecystectomy and irrigation drainage for abscess-forming perforated cholecystitis 2 years previously. His medical history was notable for gastric cancer requiring a distal gastrectomy 26 years ago. The physical examination upon admission revealed right hypochondrial pain and rebound tenderness. Laboratory testing revealed the following: white blood cell count, 11,700 /μL; C-reactive protein, 0.04 mg/dL; aspartate transaminase, 22 IU/L; alanine transaminase, 28 IU/L; alkaline phosphatase, 364 IU/L; and total bilirubin, 0.9 mg/dL. CT identified a dilated remnant gallbladder with an abscess under the abdominal wall (Fig. 3a). A small stone was noted in the cystic duct (Fig. 3b). Therefore, we diagnosed the patient with remnant cholecystitis.

**Fig. 4:**
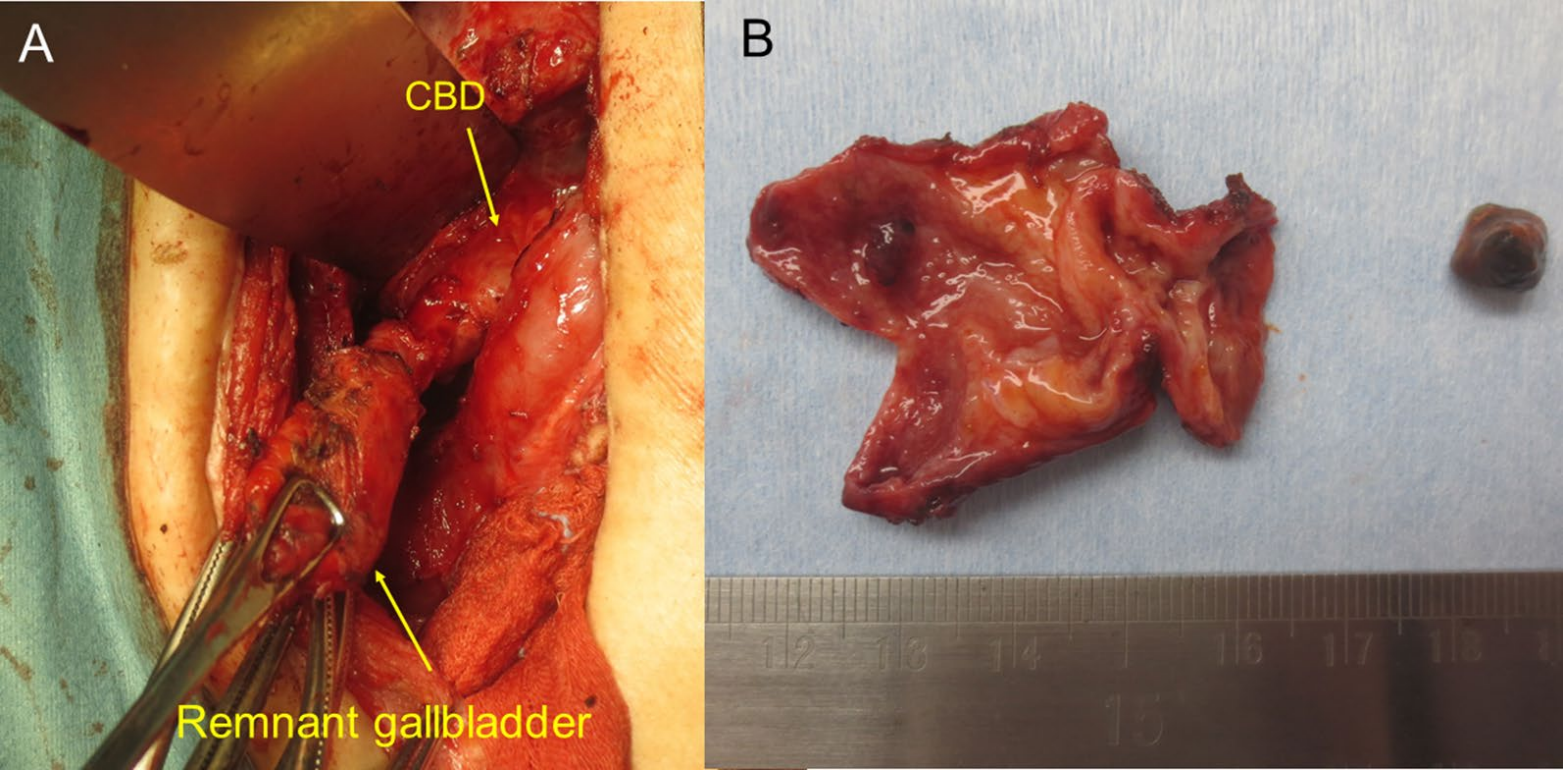
Intraoperative images of case 2. **a** Intra-operative image. The remnant gallbladder was separated from the surrounding tissue and common bile duct. **b** Specimen photography. There was no evidence of malignancy in the remnant gallbladder

Pre-procedure antibiotics were administered (cefoperazone-sulbactam [2 g × 7 days]), followed by EBS and EGBD tube placement (Fig. 3c). Elective surgery was performed 4 months after EGBD tube placement, at which point the patient had an improved white blood cell count of 5100 /μL and C-reactive protein of 0.05 mg/dL. The laparotomy was carried out via upper median incision. We were able to separate the remnant gallbladder from the surrounding tissue, allowing for identification of the cystic duct and common bile duct. After the cystic duct was ligated and cut off near the confluence of the three ducts, we completely removed the remnant gallbladder (Fig. 4a). The operative time and blood loss were 161 min and 40 ml, respectively. Histopathological examination was negative for malignancy in the remnant gallbladder (Fig. 4b).

The patient was discharged on post-operative day 7 without any complications.

## Discussion

Subtotal cholecystectomy is useful for avoiding bile duct injury when a severe inflammation in the neck of the gallbladder limits surgical views [2]. Henneman et al. reported only one bile duct injury in 625 laparoscopic subtotal cholecystectomies [4], suggesting that subtotal cholecystectomy may be useful as an aspect of reducing the risk of bile duct injury. In contrast, long-term complications associated with remnant gallbladder have been reported [3–6]. Indeed, Kohga et al. [3] reported that 1 of 35 patients (3%) who underwent subtotal cholecystectomy developed remnant cholecystitis. Between January 2018 and December 2019, 359 patients underwent LC in our hospital. We performed subtotal cholecystectomy in 9 patients (laparoscopic procedures in 2 patients, and open laparotomies in 7). None of the 9 patients had fallen stones, but one patient (Case 2 [11%]) had remnant cholecystitis. These results suggest that remnant cholecystitis is one of the most crucial complications after subtotal cholecystectomy.

Two methods of subtotal cholecystectomy have been proposed: fenestrating and reconstituting [5]. Fenestrating requires as much removal of the gallbladder wall as possible, leaving the neck of the gallbladder, and extracting any stones. The cystic duct is closed from the inside with a purse-string suture and the lowest part of the gallbladder is left open. Reconstituting also involves removing the gallbladder wall, but leaves behind the lowest part of the gallbladder, and then the stones are removed. The gallbladder wall is then closed with sutures or staples, creating a dead space that could allow for future stone formation. Although fenestrating has been associated with more postoperative bile leakage than reconstituting, most cases of bile leakage are absorbed spontaneously [4, 6]. On the other hand, the incidence of residual stones in the common bile duct was significantly higher in patients who underwent reconstituting compared with patients who underwent LC (16.6% vs 0.7%) [3]. Furthermore, in long term follow-up after subtotal cholecystectomy, reformation of gallstones was observed in up to 5% of cases and these patients usually underwent subtotal reconstituting procedures [4]. Therefore, it is necessary to be vigilant for long-term complications associated with the reconstituting procedure. In case 1 we could not perform a total residual cholecystectomy due to severe adhesions, thus we opted to proceed with reconstituting. Given his history of a mesh repair for a previous ventral incisional hernia, we were concerned that any potential post-operative bile leakage would be a nidus for a mesh-related infection, and this further supported our decision against a fenestrating procedure. Because of the potential risk for cholecystitis recurrence in the remnant gallbladder, the patient will have long-term, close follow-up evaluation.

Further studies are needed to assess the operative indications for fenestration versus reconstitution.

Our two cases suffering remnant cholecystitis underwent reconstituting procedures, and stones were observed in the remnant gallbladder. Kohga et al. measured the remnant gallbladder diameter by MRCP in 35 patients after subtotal cholecystectomy [3]. No long-term complications were observed in 15 patients in whom the remnant gallbladder could not be identified by the image. On the other hand, long-term complications were observed in 8 of 20 patients (median remnant gallbladder diameter, 22.6 mm) in whom the remnant gallbladder was identified on imaging. They reported that there was a significant correlation between the remnant gallbladder diameter and long-term complications. Due to the size of the remnant gallbladder in both our cases, 38 × 36 mm and 47 × 29 mm, respectively, they had a higher risk for long-term complications.

Remnant cholecystitis is thought to be due to the severe adhesions from both prior cholecystitis and current inflammation. Moreover, there may be some anatomical variations caused by the previous surgery. Therefore, the surgical difficulty for remnant cholecystitis is considered high and bridging prior to surgery may be needed to minimize potential complications associated with the high inflammatory state. Percutaneous transhepatic gallbladder drainage (PTGBD) is often performed to improve the inflammation for severe cholecystitis as recommended in TG18. However, PTGBD for remnant cholecystitis is often difficult because the remnant gallbladder consists of the lowest part of the gallbladder and the area of remnant gallbladder contacting with the gallbladder bed is relatively small. EGBD is a useful technique to drain remnant gallbladder without requiring transhepatic puncture [7–9]. In fact, although both of our cases had remnant gallbladders greater than 30 mm, it was impossible to perform PTGBD. EGBD improved the persistent cholecystitis, allowing for elective surgery afterwards. EGBD is known as a difficult procedure. In our hospital, we attempted to perform EGBD on 47 patients with cholecystitis (not remnant cholecystitis) from December 2011 to December 2018. Among the 47 patients, 4 patients failed (2 patients had extravasation from the cystic duct; 1 patient had impaction of stones in the cystic duct; and it was not technically possible to cannulate the cystic duct in 1 patient) and EGBD was successful in 43 patients (91.5%). Therefore, outcomes for patients with remnant cholecystitis may be improved if transferred to advanced facilities with not just experienced surgeons, but also endoscopists trained in EGBD tube placement techniques. Moreover, it may be difficult to identify the remnant gallbladder due to severe adhesion or anatomical variation, in which case the EGBD tube could be a landmark to identify the remnant gallbladder intraoperatively.

## Conclusion

We describe two cases of remnant cholecystitis for which residual cholecystectomy was performed after subtotal cholecystectomy. Since surgery for remnant cholecystitis is expected to be very difficult, pre-surgical management with EGBD tube placement may allow for improved outcomes.

## Data Availability

The dataset supporting the conclusions of this article are available in the manuscript.

## Abbreviations

LC: Laparoscopic cholecystectomy
TG18: Tokyo 2018 Guidelines
CT: Computed tomography
MRI: Magnetic resonance image
EBS: Endoscopic biliary stent
EGBD: Endoscopic gallbladder drainage
PTGBD: Percutaneous transhepatic gallbladder drainage
